# Supplementary material for: Evolutionary Dynamics of Foot and Mouth Disease Virus Serotype A and Its Endemic Sub-Lineage A/ASIA/Iran-05/SIS-13 in Pakistan
Source: Viruses. 2022 Jul 26;14(8):1634. doi: 10.3390/v14081634 (PMC9331208; doi:10.3390/v14081634)
Supplement: Supplementary file 1 [file viruses-14-01634-s001.zip › viruses-1740966-supplementary.pdf]

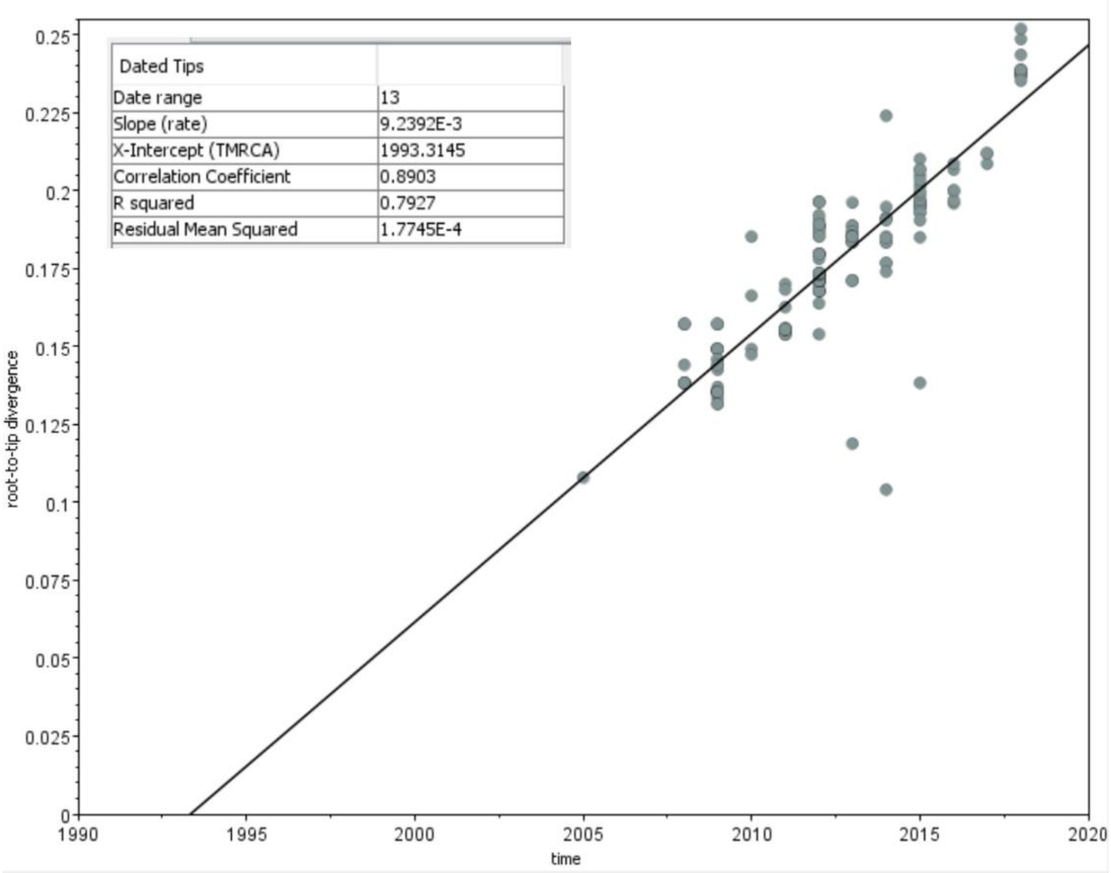

Figure S1. Regression plot of root-to-tip divergence with time in years. The unrooted, maximum-likelihood tree generated in IQtree was visualized in TempEst for temporal signal. Best-fitting root was selected for the tree and the heuristic residual mean squared function was used for regression analysis of divergence with time. R squared value 0.79 showed a good fit of the regression model and hinted at a strong temporal signal in sequence data.

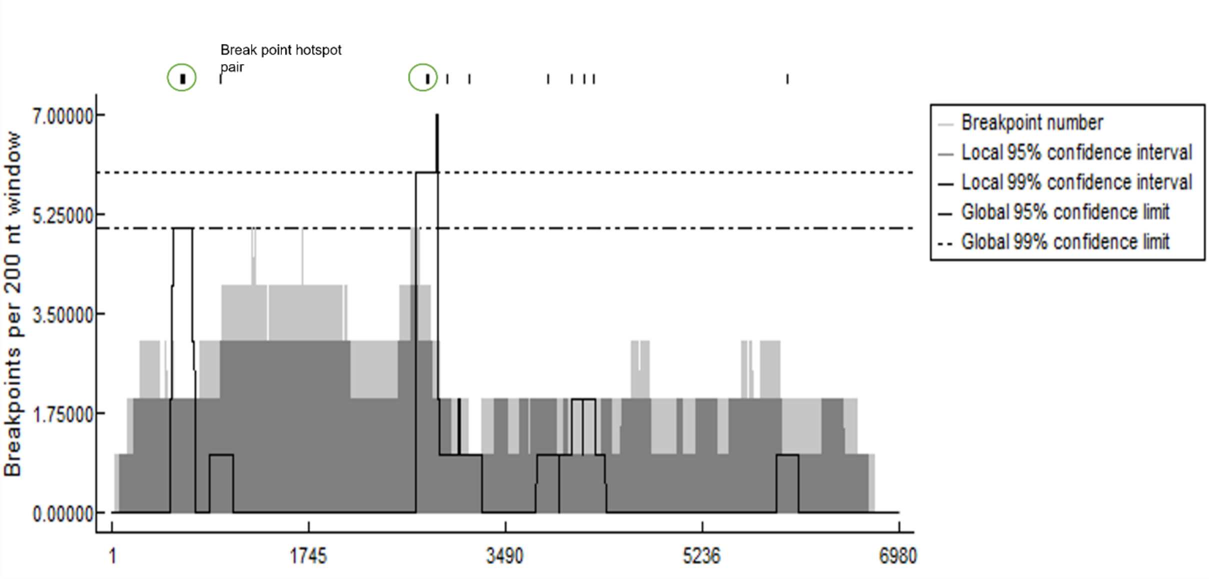

Figure S2. Recombination Breakpoints position inferred for complete genome sequences of three serotypes O, A, and Asia1 of FMDV in RDP4. The breakpoint hot spot pair indicates if a breakpoint has occurred at ~ 900 positions then the other will occur at ~ 2300. The area between these two breakpoints is seemingly a cold spot where recombination is less likely to occur.

Table S1. List of accession numbers of complete genome sequences s of type A, O, and Asia-1 from Pakistan, available in NCBI GenBank.

Accession #	Isolate ID
OM455480.1	A/PAK/ICT/1/2008/ESF-10
OM455482.1	A/PAK/KCH/7/2009/ESF-10
KY446902.1	A/Hafizabad/QOL-UVAS-Pak/2005
MN062587.1	A/SIN/PAK/L693/2009/BAR-08
EF494487.1	A/isolate/PAK1/2006/Iran-05
EF494488.1	A/PAK5/2006
OM455476.1	A/PAK/ICT/231-1/2012/HER-10
OM455481.1	A/PAK/ICT/276/2012/FAR-11
OM455465.1	A/PAK/ICT/7-3/2012/HER-10
JN006722.1	A/SIN/PAK/L4/2008/SIN-08
OM455466.1	A/PAK/ICT/008-3/2012/HER-10
OM455477.1	A/PAK/ICT/237-1/2012/HER-10
OM455471.1	A/PAK/ICT/168-3/2012/HER-10
OM455467.1	A/PAK/ICT/059-1/2012
OM455472.	A/PAK/ICT/170-1/2012/HER-10
OM455475.1	A/PAK/ICT/229-1/2012/HER-10
OM455474	A/PAK/ICT/208-1/2012/HER-10
OM455484	A/PAK/SGD/12/2012/HER-10
OM455483.1	A/PAK/PSH/34/2012/HER-10
OM455479.1	A/PAK/FSD/4/2012/HER-10
OM455478.1	A/PAK/ICT/238-1/2012/HER-10
OM455473.1	A/PAK/ICT/177-4/2012/HER-10
OM455468.1	A/PAK/ICT/131-4/2012/HER-10
OM455469.1	A/PAK/ICT/149-4/2012/HER-10
OM455470.1	A/PAK/ICT/168-2/2012/HER-10
MZ493233.1	FMDV/A/ASIA/Iran-05/SIS-13
MZ493234.1	FMDV/A/ASIA/Iran-05/SIS-13
MZ493232.1	FMDV/A/ASIA/Iran-05/SIS-13
MN062587.1	FMDV/O/SIN/PAK/L693/2009
GU384683.1	FMDV/O/PAK/45/2008
JN006719.1	As/SIN/PAK/L5/2008
JN006720.1	As/SIN/PAK/L2810/2008
KT003716.1	FMDV/O/ Nari/UVAS-Pak/2005
KY446901.1	FMDV/Asia1/Mazbi/QOL-UVAS-Pak/2006
KY446903.1	FMDV/O/FBD/QOL-UVAS-Pak/2005
MH784403.1	FMDV/O/PAK/17/2017
MH784404.1	FMDV/O/PAK/4/2017
MH784405.1	FMDV/O/PAK/14/2017
MN062581.1	FMDV/ O/PUN/PAK/L1358/2009
MN062582.1	FMDV/O/PUN/PAK/L1370/2009
MN062583.1	FMDV/O/ISL/PAK/L1412/2009
MN062584.1	FMDV/O/NWF/PAK/L1417/2009
MN062587.1	FMDV/A/SIN/PAK/L693/2009
MN953619.1	FMDV/O/PAK/1/2019
MN953620.1	FMDV/O/PAK/2/2019
AY593795.1	FMDV/asia1-1/pak iso3/1954
